# Review of Epidata Entry and Analysis Freewares

**DOI:** 10.4103/0970-0218.45384

**Published:** 2009-01

**Authors:** Shavinder Singh

**Affiliations:** Department of Community Medicine, Christian Medical College, Ludhiana - 141 008, India. E-mail: sandhuss@rediffmail.com

Sir,

Database management and data analysis are important skills of public health professionals. Available epidemiological and statistical softwares can be divided broadly into two categories; commercial and open source products like other computer softwares. SPSS, SAS and STATA represent the former category while Epidata entry, Epidata analysis, Openepi and Epiinfo DOS (EPI6) and Windows, belong to the latter category.

EPI6(1) users remember its functionality and range of commands and capabilities. The underlying concept is solid. Type your questionnaire and use the specific variable types. Convert into a record file and begin the data entry. The questionnaire, record, and check files are known as qes, rec, and chk files, respectively. The records file is analysed in the analysis program of EPI6.

Criteria for the appraisal of statistical softwares for epidemiology(2) include not only its capabilities, but also “smoothness of installation, simplicity of interface, ease of use, completeness and statistical quality of the documentation, completeness and appearance of statistical graphics, accuracy of statistical computations. Using these criteria two useful freewares has been evaluated.

**Epidata entry(3):** Jens Lauritsen developed it preserving the qes/chk/rec functionality of original EPI6. The installation is simple. It has standard as well as user defined setup for installation into a specific hard disk or portable drive. The steps of creating a database are: Type a questionnaire, create a database file, enter data and analyse in Epi Analysis freeware. The look-up lists can be embedded in the main database file. Database file can be documented and also exported to other software file formats such as text, dBase III, Excel, Stata, SPSS, and SAS. Epidata entry 3.1 is a stable and robust relational database management system. Window ease of use, mouse, fonts and colours are additional features to facilitate the data entry. A help manual is available from the website.

**Epidata analysis(3):** It is a separate data analysis freeware. Its installation is smooth and can be installed on any drive (Including USB pen drive) using user-defined settings. The basic commands are same as that of EPI6 with enhanced features and switches for fine-tuning of the output. The user interface has both command line and dialogue boxes.

For experienced EPI6 users, command line is easier to use. It includes most popular statistical routines for general statistical analysis, complete graphics and reporting capabilities. The list of commands can be seen by F2 and variables by F3 keys, as was the case in EPI6. The dialogue boxes of FREQ, TABLE, MEANS and GRAPH commands are exhaustive. The tables command has been refined with additional switches to fine-tune the output such as row or column percentages, specific statistical test output and outbreak analysis parameters. Frequency, cross and stratified tables, with features of arranging the total percentages in the ascending or descending order, has been provided. In addition, StatTables command can produce weighted data such as counts, averages, mean, standard deviation and percentiles that are great features from a freeware. You can get what you want by clicking specific options in the dialogue box.

It also performs descriptive statistics, Chi square test, Student *t*-test, one-way ANOVA, Kruskall Wallis, Fisher's exact test, correlation and regression. The American and European date selection syntax is different from EPI6.

The graphs such as bar, pie, histogram, scatter, and SPC charts have been given a face-lift. These graphs can be copied and pasted on the slides or text document. The output can be in the form of text or html files which can be edited in the standard window word processors. A command reference can be accessed from within the software.

Epidata entry and Epidata analysis are good tools for the low resource projects, post-graduate thesis and even developing permanent database applications. These can be installed on to USB pen drive using the user defined settings and run directly. Epidata Entry has been tested on the Linux (Suse and Fedora core variants) also using WINE software. An on-line list of users is there to handle any queries.

**Limitation of the product:** Correlation and regression can be performed up to 5000 records file only.
